# Competency scale of quality and safety for greenhand nurses: instrument development and psychometric test

**DOI:** 10.1186/s12912-024-01873-5

**Published:** 2024-03-29

**Authors:** Run Nan Zhai, Ying Liu, Jia Xin Wen

**Affiliations:** School of Nursing, Dalian Medical University, No 9 Western Section South LvShun road, 116044 Dalian city, Liaoning province China

**Keywords:** Competency, Quality, Safety, Instrument development, Nurses

## Abstract

**Background:**

Guaranteeing nursing service safety and quality is a prioritized issue in the healthcare setting worldwide. However, there still lacks a valid scale to measure the quality and safety competencies of newly graduated nurses globally.

**Methods:**

This scale was developed in two phases. In Phase One, a literature review and three-round e-Delphi were conducted to generate the initial item pool; while in Phase Two, five experts tested the content validity of the scale. The construct validity was evaluated using confirmatory factor analysis (CFA), and the data were collected among 1,221 newly graduated nursing students between May, 2017 and August, 2017. Finally, the internal consistency reliability and test-retest reliability were tested.

**Results:**

The final version’s Competency Scale of Quality and Safety (CSQS) was confirmed by the CFA involving 64 items in six dimensions, including patient-center care, safety, evidence-based practice, collaboration and teamwork, continuous quality improvement, and informatics. The results of data showed that the data supported the modified model of CSQS (Standardized Root Mean Square Residual = 0.03, *p* = 0.053, Adjusted Goodness of Normed Fit Index = 1.00, Root Mean Square Error of Approximation = 0.007, Fit Index = 0.95, Goodness of Fit Index = 0.97, χ^2^/df = 1.06), and the standardized factor loadings of items were from 0.59 to 0.74 (*p* < 0.05). The internal consistency reliability of the total scale was 0.98, and the test-retest reliability was 0.89.

**Conclusions:**

CSQS was a valid and reliable instrument to measure the safety and quality abilities of greenhand nurses, and could be fully utilized by nursing students, greenhand nurses, nursing educators, as well as hospital nursing managers.

**Supplementary Information:**

The online version contains supplementary material available at 10.1186/s12912-024-01873-5.

## Background

At the beginning of the 21st-century, deficiencies of healthcare safety and quality were highlighted by the Institute of Medicine [1]. One of the primary aims of healthcare organizations is to furnish patients with high-quality and safe care [2]. This imperative stems from reports indicating that millions of patients worldwide suffer disability or death each year due to unsafe medical practices. Moreover, caregiver infractions against patient safety can impose significant financial burdens on patients [3]. Upholding the purpose to protect medical care deficiencies, a fresh competency framework for all health careers to provide patient-centered care and improve the safety and quality of patients’ care was put forward by the IOM in the early 2003 [4]. Nurses are pivotal in the healthcare service system, constituting the foremost group in accomplishing the goal of providing quality and safe care to patients [2].However, the nursing errors or deficiency remain high among newly graduated nurses. For instance, 86 nursing errors were reported from 42 new nurses by Zhang [5]. A study by Treiber and Jones also revealed that approximately 55% of newly admitted nurses committed nursing errors within the initial five years of their clinical careers [6]. Thus, the competency based quality and safety education for nurses has been correspondingly developed, which is consistent with the mission of IOM [7]. In the early 2005, the initiative was launched to establish the national competency framework for Quality and Safety Education for Nurses (QSEN) by the reputed organization of American Association of Colleges of Nursing (AACN) [1].The QSEN framework was respond to quality and safety concerns by providing the foundational knowledge, skills and attitudes to ensure that nurses provide quality and safe care in their daily practice [8]. Furthermore, over the past decade or so, this competency-based framework has been integrated into the curricula of nursing programs in numerous countries [9].

McClelland, an American psychologist, initially referred competency to the knowledge, attitudes, skills and traits that affected the job performance of an individual [10]. The globally renowned Knowledge, Skills, and Attitudes (KSA) model, proposed by Benjamin Bloom [11], has found widespread application in the human resource management industry for assessing employees’ competency [11, 12]. In the field of nursing, the competencies of QSEN involve a complete set of knowledge, skills, and attitudes as the chief tactics that effectively apply competencies including patient-center care, safety, evidence-based practice, collaboration and teamwork, continuous quality improvement, and informatics [7], which is based on KSA model [9]. The QSEN competency framework was initially developed for prelicensed nursing students with the purpose of establishing minimum standards for secure clinical settings safety and high-quality practice [7]. However, the development of the comprehensive QSEN competency assessment scale to assess graduated bachelor nursing students (BNS) experienced a long history upon the completion of this study.

At the beginning, Cronenwett, et al. [7] recruited 16 universities among the USA to clarify the definitions associated with the six dimensions of QSEN ability components and the guidelines of overall teaching courses for prelicensure nursing students at graduation. Soon after, Barton, et al. [13] performed a Delphi among the USA for consensus regarding varied QSEN abilities to be included in teaching courses. At last, a total of 162 QSEN abilities were obtained from 18 nursing experts among 16 states. Simultaneously, Sullivan, et al. [14] conducted a survey in 17 universities among 565 students to develop a nursing students assessment tool regarding the preparation and importance of these QSEN abilities. This tool was named as QSEN of student evaluation survey (QSEN-SES), which included 19 items of learned contents in the knowledge dimension; while in the skill dimension, it was composed of 22 items of skill preparedness; and in the attitude dimension, it was comprised of 22 items. Additionally, the results confirmed that QSEN competencies were important for the further work of most of the nursing students. However, this scale did not test the psychometric property.

Later, Pauly-O’Neill, et al. [15] conducted a study to assess the QSEN competencies of BNS using the observational checklist tool, but there was also no psychometric property reported for this tool. At the same time, Piscotty, et al. [16] developed BSN Quality and Safety Self-Inventory (QSSI) with two dimensions and 18 items. Although QSSI featured acceptable validity and reliability, it failed to reflect the QSEN framework through six components.

The utilization of the QSEN framework was not only implemented in the USA, but also in the South Korean. Lee, et al. [17] assessed the evaluation methods and contents of the QSEN competencies of nursing students. They translated and modified the QSEN-SES of Sullivan, et al. [14] into a Korean version. However, the Korean version of QSEN-SES only reported the content validity, and there was no other psychometrics report.

Additionally, Nygårdh, et al. [18] applied the QSEN framework to create the competency tool of QSEN in Sweden. According to previously reported results, three main dimensions were chosen to present the QSEN competencies by Nygårdh, et al. [18]. The reliability of the Swedish version of the QSEN instrument was compared with the reliability of the instrument of Cronenwett et al. [7], but its validity and internal consistency reliability were not evaluated.

More recently, based on the QSEN framework, Liu, et al. [19] conducted an e-Delphi study in China to develop indicators for evaluating the QSEN competencies of BNSs at their graduation. Through three rounds of e-Delphi with 22 experts, consensus was achieved regarding 88 indicators in six dimensions among these experts that a comprehensive indicator could be used to formulate the curriculum and teaching content, while it could not be used as the assessment tool to measure newly graduated nurses’ quality and safety competencies (QSC). Therefore, further efforts were required to develop the assessment tool.

Through the history of QSEN competency development, it can be noticed that various countries have recognized the importance of formulating the QSEN competencies of nursing students to provide safety and quality nursing care to patients in their future work. However, there is an obvious deficiency between the requirement of an assessment tool and QSEN [20]. Besides, in the nursing discipline, the students achieving baccalaureate are regarded as the leading workforce in the clinical practice [21], but no Competency Scale of Quality and Safety (CSQS) has been found to measure their QSC when they are graduated, making it necessarily important to develop an assessment tool to evaluate the QSC of the pre-licensed BNSs using the QSEN framework. To this end, the present study was carried out to develop the evaluation scale and measure the QSC of BNSs upon their graduation.

## Methods

### Research design and procedures

**Fig. 1:**
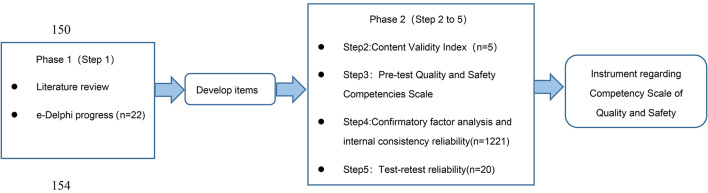
The development and psychometrical testing process regarding the competency scale of quality and safety

The cross-sectional survey design was conducted to develop and test the scale psychometrical properties in two phases. Literature review and three-round e-Delphi were conducted for the pool development of the CSQS items in the first phase, while in the second phase, the psychometrics of CSQS were assessed through Step 2 to Step 5 in Fig. 1.

### First Phase: CSQS items development

Step 1: Based on the QSEN framework, the item pool was generated through literature review and three-round e-Delphi among 22 nursing experts [19]. The initial round of e-Delphi sought to elucidate the concept and components of QSC-BNS by soliciting insights from experts through two semi-structured open-ended questions. These questions were “How do you define the QSC-BNS?” and “How do you define each dimension of the QSC-BNS?“. Following content analysis from experts’ opinions and the integration of literature review items, a total of 89 items across six dimensions were identified, including patient-centered care, safety, evidence-based practice, collaboration and teamwork, continuous quality improvement, and informatics, were identified. Subsequently, these items were presented to the same panel of experts for the second round of e-Delphi, where they were asked to evaluate the importance of each item using a 5-point Likert scale. Items meeting the criteria of an interquartile range (IR) of ≤ 1.5, a median of ≥ 3.5, and a consensus level of agreement (CLA) of ≥ 70% were identified to have reached the consensus level and were retained, following the approach suggested by Keeney et al. [22]. One item failed to achieve a CLA of more than 70% and was consequently removed. The remaining 88 items were then resubmitted to the same group of experts to obtain their consensus opinion. At the end of the third round e-Delphi, consensus was achieved among 88 items with a median ranging from 4.00 to 5.00; the IR ranged from 0.00 to 1.50, and the CLA ranged from 76 to 100%. Thus, the initial item pool of CSQS was composed of 88 items in six dimensions, including patient-centered care (13 items), safety (17 items), evidence-based practice (15 items), collaboration and teamwork (15 items), continuous quality improvement (13 items), and informatics (15 items).

The CSQS was a five-point Likert scale with an evaluation score of 1 = completely incompetent to 5 = completely competent, with a lower score indicating a lower QSC, and a higher score suggesting a higher QSC.

### Second phase: CSQS psychometrical testing

#### Testing content validity

Step 2: The content validity index was defined as the ratio of scale items scored as quite or very relevant to constructs by all of raters [23]. The CSQS content validity with 88 items were tested among five experts. The experts inclusion criteria included: (1) those who at least obtained a master degree or taught master students; (2) those who taught nursing management courses or had experience of working in management positions; and (3) those who would like to assess items. Four-point Likert Scale (from 1 = not relevant to 4 = very relevant) was utilized by experts to assess items relevant to defining the constructs [23]. Based on the suggestions proposed by Polit, et al. [24], the Content Validity Index (I-CVI) and CVI of scale average (S-CVI/Ave) of the items equal or greater than 0.8 and 0.9 were acceptable when there were five experts.

#### Pre-testing CSQS

Step 3: The pre-testing of CSQS was conducted among 31 BNSs to identify the clarity, difficulties, and comprehensiveness of the scale. These students met the inclusion criteria: (1) those who had entranced a bachelor nursing program; and (2) those who had completed the national nursing license examination at their graduation. The exclusion criteria was nursing students who failed to finish the coursework or take the nursing license examination.

#### Testing factor structure and reliability of CSQS

Step 4: Based on the instrument development progress by Crocker and Algina [25] and DeVellis [26], direct confirmatory factor analysis (CFA) without exploratory factor analysis (EFA) can be feasibly conducted when the theory can support the instrument development. It has become popular to directly conduct CFA without EFA in contemporary instrument development progress, such as the development of Medication Adherence Scale [27] and Readiness for Practice Instrument for Senior Undergraduate Nursing Students [28]. Herein, the development of CSQS was strongly based on the conceptual framework of QSEN from the beginning to the end. Additionally, QSEN specifically included six dimensions of QSC [7], so that the structure testing of CSQS used the CFA directly and excluded the EFA method. This is attributed to the fact that EFA can only group items [26], failing to clarify the structures of CSQS based on the QSEN framework. However, determined by the theory [26], CFA can clarify the structures of instruments. In this case, the CSQS was hereby evaluated using the CFA method, and the internal consistency reliability (ICR) of CSQS was reported by Cronbach’s alpha.

Step 5: The final version of CSQS stability was evaluated by test-retest reliability [23]. Twenty BNSs were included in this process with 2 weeks of interval [25]. The inclusion and exclusion criteria for the BNSs were the same as described in Step 3.

#### Participants and settings

According to Kline [29] in the progress of constructing validity testing, one item involving 10 to 20 participants was recommended. Herein, one item involving 15 participants were used to determine the sample size. Considering the attrition of participants, 10% of the sample size was added. Thus, a total of 1,452 participants were required. The inclusion and exclusion criteria for selecting the participants were the same as described in Step 3, who enrolled in a bachelor nursing program, fulfilled the required courses, and successfully passed the national nursing license examination upon graduation were included in the study. Initially, the purposive sampling method was employed to identify universities offering bachelor nursing programs. Subsequently, nine universities across seven cities in China, with which the researcher could establish connections, were selected for inclusion. Following this, a convenient sampling strategy was utilized to select bachelor of nursing students who met the predetermined inclusion and exclusion criteria from these universities.

### Data collection and ethical consideration

The ethical application of this study was approved by Ethical Committee of researcher Organization (No. NYHL1701), and permissions for the data collection were received from each university. The data collection process was composed of the following steps: (1) the researcher assistants in each university were informed of the study purpose, as well as the exclusion and inclusion standards for the participants; (2) the researcher assistants and researcher sent the information sheet, consent form, and package of questionnaires to BNSs by survey host of “So Jump” survey host or Mail; and (3) the researcher checked the completeness of the answered questionnaires. The data were collected from May 2017 to August 2017.

### Statistical analysis

The frequencies, percentages, standard deviations (SD), median and means of the participants were described using IBM SPSS 22.0 program. SPSS software was also utilized to analyze the skewness, kurtosis and item-to-total correlation (ITC) of the items before performing CFA. The Cronbach’s alpha greater than 0.8 was taken to define the acceptable ICR of final version of CSQS.

The researcher conducted the CFA using the LISREL 8.72 program to test the CSQS factor structures. Before operating the CFA, the normality, multicollinearity and linearity were tried out using the SPSS software. The consistency between the measurement model and the research data was assessed by standards adopted from Hair., et al. [30], including Root Mean Square Error of Approximation (RMSEA) < 0.08, *p* ≥ 0.05, Goodness of Fit Index (GFI) > 0.90, Standardized Root Mean Square Residual (SRMSR) < 0.07, Adjusted Goodness of Fit Index (AGFI) > 0.90, Normed Fit Index (NFI) > 0.90, and χ^2^/ df < 2.00. In the case of a sample size greater than 500, the cut-off factor loading of the item above 0.3 was considered suitable [30]. Herein, the significant α was set as 0.05.

## Results

### Characteristics of the participants

A total of 1,452 questionnaires were distributed, and 1,232 completed questionnaires were gathered successfully. After excluding univariate outlier with absolute z-score more than 3 [29], 1,221 questionnaires were adopted, with a response rate of 84.09%. The participants were selected from 9 schools, involving 76 boys (6.2%) and 1,145 girls (93.8%) aged from 20 to 25, with a mean score of 22.70 (SD: 0.95).

### Psychological testing of CSQS

#### Content validity

Herein, the CSQS S-CVI/Ave was 0.98, and the I-CVI ranged from 0.8 to 1 among five experts. Both met the standards recommended by Polit, et al. [24]. Therefore, the CSQS content validity was contented. In addition, the wording of some items was revised following the opinions from the experts.

#### Pre-test CSQS

In Step 3, 31 BNSs did not have any problems regarding the description of the items. The Cronbach’s alpha of each dimension ranged from 0.89 to 0.94, while that of the overall scale was 0.98.

#### Construct validity and internal consistency reliability

The items were hereby analyzed before running CFA among 1221 BNSs. According to the descriptive statistical results, the kurtosis value of 88 items ranged from − 0.54 to 0.40, while the skewness value was between − 0.46 and − 0.11. Since the acceptable skewness values was between − 1 and + 1 [31], and kurtosis absolute values was under 2 [32], all the items met the selection criteria for further analysis. Additionally, the score of ITC was between 0.54 and 0.74 in this study. Ferketich [33] stated that an ITC smaller than 0.30 did not contribute much to the measurement concept, while an ITC larger than 0.70 might be redundant [25]. Thus, the researcher removed items with an ITC less than 0.30 or greater than 0.70. After analysis, the scores of 24 items were above 0.70, and were deleted. The ICR values of the total scale were 0.98. Additionally, the ICR values of the six CSQS dimensions ranged from 0.87 to 0.93, all above 0.8 [23] (Table 1), justifying the reasonability of conducting the CFA.

**Table 1:** The Competency Scale of Quality and Safety internal consistency reliability (*N* = 1221)

Dimensions	Cronbach’s α	Items number
Patient-centered care(PCC)	0.93	13
Collaboration and Teamwork(CAT)	0.93	14
Evidence-based practice(EBP)	0.88	7
Continuous quality improvement(CQI)	0.87	7
Safety (SAF)	0.92	11
Informatics(INF)	0.93	12
Total	0.98	64

**Fig. 2:**
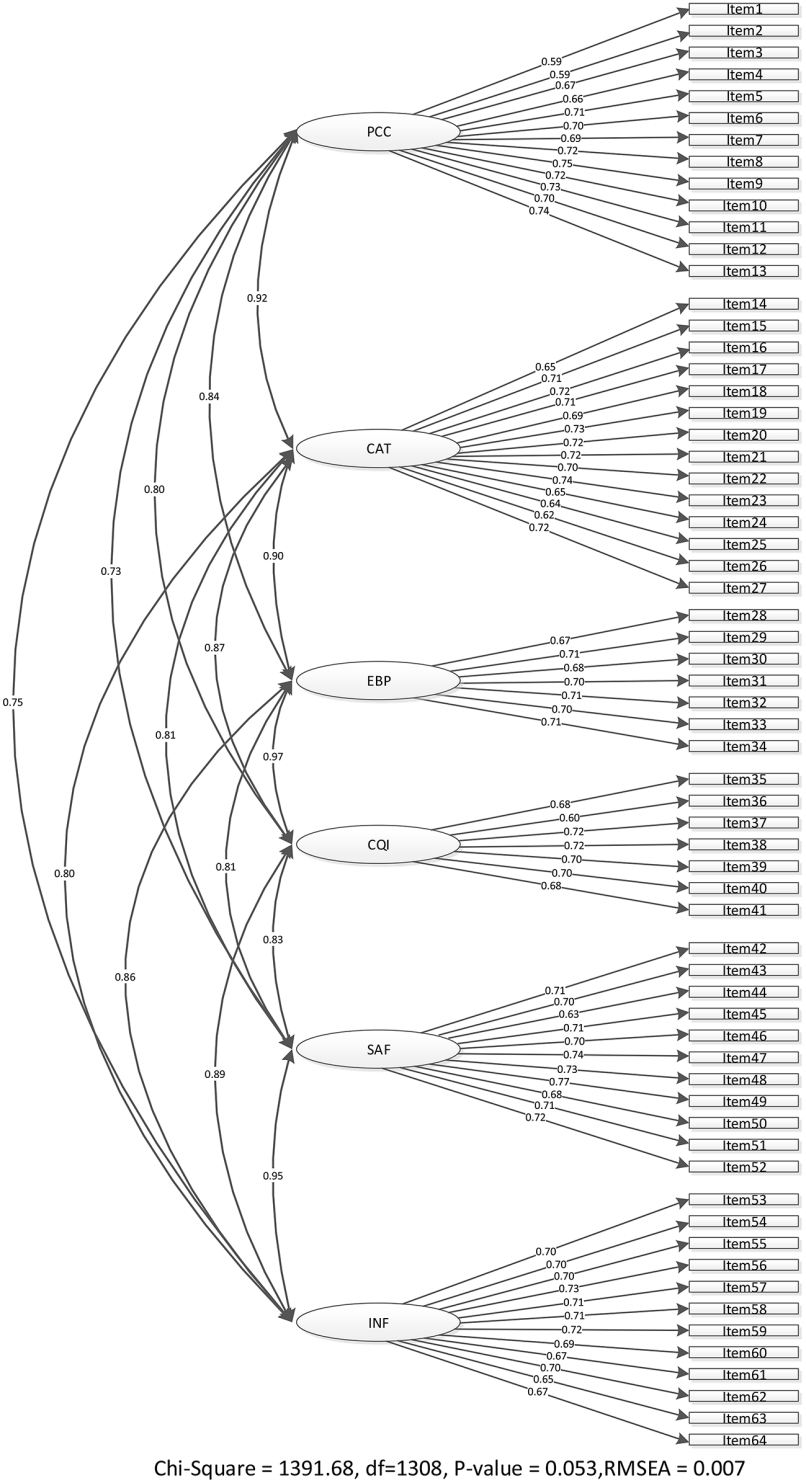
The measurement model regarding the final competency scale of quality and safety. *Note* CQI = Continuous quality improvement, CAT = Collaboration and Teamwork,PCC = Patient-centered care, SAF = Safety, EBP = Evidence-based practice, INF = Informatics, RMSEA = root-mean-square error of approximation

Three assumptions were tested before performing CAF, and these assumptions were found acceptable. The maximum likelihood estimation method was adopted for implementing CFA. Six dimensions with 64 items were included in the modified CSQS, and the outcome is shown in Fig. 2. The GFI indicators of the modified CSQS were satisfied: SRMSR = 0.03, *p* = 0.053, AGFI = 0.95, RMSEA = 0.007, GFI = 0.97, NFI = 1.00, and χ^2^/df = 1.06. Besides, the standardized factor loadings of items were from 0.59 to 0.74 (*p* < 0.05). The detailed information of each item is presented in Additional file 1. The mean scores for each dimension are delineated in Table 2.

**Table 2:** The description of Competency Scale of Quality and Safety’ each dimension (*n* = 1221)

Dimensions	Minimum	Maximum	Mean	Standard Deviations
Safety (SAF)	2.18	5.00	3.89	0.57
Collaboration and Teamwork (CAT)	2.21	5.00	3.83	0.54
Patient-centered care (PCC)	2.23	5.00	3.83	0.55
Informatics (INF)	2.00	5.00	3.78	0.58
Evidence-based practice (EBP)	1.86	5.00	3.70	0.62
Continuous quality improvement (CQI)	1.86	5.00	3.62	0.62

#### Test-retest reliability

In Step 5, the researchers tested the stability of the final version of the CSQS. The CSQS questionnaires were sent to 20 BNSs, and the time interval between the first and the second to answer the questionnaires were 14 days. The results showed the test-retest Pearson’s product moment r of each dimension was between 0.72 and 0.89 and the total scale was 0.89. Since the reliability coefficient of the total scale was higher than 0.80 as recommended by Polit and Beck [23], the CSQS stability was considered favorable.

## Discussion

In the current clinical environment, it is of great value to keep the quality and safety of healthcare services. Based on the QSEN competency framework and the common view of Chinese experts, six dimensions of QSC were hereby formed, which were congruent with previous academic claims [7, 18]. Additionally, it was also known as the first valid and reliable scale developed globally to measure the QSC of newly graduated nurses with bachelor degree.

The development of CSQS items ensued from meticulous scrutiny, integrating insights derived from an extensive literature review and three iterative rounds of e-Delphi. During the initial e-Delphi round, definitions pertaining to QSC and its six dimensions were distilled from semi-structured questionnaires, drawing upon the expertise of Chinese professionals and aligning with the QSEN competency framework [7, 8]. Subsequent rigorous evaluations across the second and third e-Delphi rounds yielded a refined set of 88 items, meeting stringent criteria (IR ≤ 1.5, median ≥ 3.5, and CLA ≥ 70%) [22]. This meticulous process secured agreement from Chinese experts, affirming the suitability of these items for evaluating the quality and safety competencies of BNSs.

In the second phase, the psychometric properties of CSQS were tested through four steps including CVI testing, pre-testing CSQS, CFA testing, and test-retest reliability. The purpose of conducting CVI testing was to assess the ability of the instrument’s items to effectively capture the specific constructs of interest [25]. With both S-CVI/Ave and I-CVI meeting the criteria outlined by Polit et al. [24], it is evident that the constructs of CSQS adequately reflect the QSC of BNSs. In order to ensure the readability of CSQS for participants, a pretesting phase was implemented. Results indicated that the items within CSQS were easily comprehensible, exhibiting an initial high level of intrinsic consistency (ICR value > 0.8) [24]. Prior to performing CFA, item analysis was conducted. This analysis identified 24 items with excessively high ITC scores (over 0.7), suggesting redundancy and the potential for participant fatigue [25, 33]. Consequently, these items were removed. Subsequently, the CFA conducted on the remaining 64 CSQS items supported the final modified model with empirical data and all items’ factor loadings exceeding 0.3 [30]. Thus, the six dimensions of the final 64-item CSQS demonstrated satisfactory construct validity. Furthermore, the ICR value of the final CSQS was notably high, with both the total scale and each dimension’s score surpassing 0.8 [23], indicating a homogeneous reflection of the overarching construct of QSC. Moreover, the stability of the final CSQS was confirmed, with the total scale’s score exceeding 0.8 [23]. Based on the average scores of the six dimensions ranked in descending order, they were discussed in an orderly manner as follows.

Safety competency is defined as the knowledge, skills, and attitudes of BNSs in the future clinical work to grasp nursing technology and knowledge, abide by hospital regulations and rules, standardize nursing operations, protect patients and themselves from injury, or minimize the risk of injury during their practice or clinical work. Nygårdh, et al. [18] also proposed that nursing students should avoid risk factors while implementing patient care, which was consistent with the present findings. Furthermore, the World Health Organization’s (WHO) World Alliance for Patient Safety endeavors to incorporate patient safety courses into curricula worldwide, with the goal of instructing medical and nursing students in risk management [34]. Hence, the enhancement of patient safety can commence with the education of students [35].

In addition, from patient admission to discharge, nurses spend the most time to communicate and contact with patients and play a key role in protecting their rights and security, which makes nurses one of the high-risk groups of occupational exposure. Thus, efforts should be made not only to ensure the safety of patients, but also the occupational safety of nurses. In this developed measurement tool, the content related to the occupational safety of nurses was included, which was consistent with the results of Qaraman, et al. [36], who also stressed the necessity of providing training concerning occupational health and safety for nursing students by setting up appropriate courses.

Additionally, in this study, safety competency garnered the highest average score among the six dimensions. This outcome could be attributed to nursing schools placing greater emphasis on patient safety education for nursing students, addressing perspectives from both patients and nurses. Educational institutions typically aim to instill safety competencies in nursing students by providing training in both patient and occupational safety [37]. Furthermore, teaching hospitals often enhance nursing students’ safety competency through the implementation of patient safety education programs [38] and occupational health training [39]. However, it’s noteworthy that the item pertaining to “Handling specialist resuscitation procedures and being capable of performing resuscitation work” received the lowest score within this dimension. Hence, there’s a pressing need to enhance students’ proficiency in resuscitation workflows and complex problem-solving skills [40].

The competency of collaboration and teamwork refers to the knowledge, skills, and attitudes of BNSs in further clinical work to collaborate with multidisciplinary team members and play their duty role in the group, such as coordinating with team members, improving team member communication, and making the decision with patients together. In addition to doctors, nurses and patients, the chief nurses, other nursing staff, patients’ families, nutritionists and cleaners should also be included as team members. This dimension was previously brought up [41]. Taking the operating room team as an example, it is crucial to improve the team cooperation ability of all members for the successful achievement of the goal. In their study, Burke et al. [42] highlighted coordination, cooperation, and collaboration as fundamental focal points essential for shaping and executing integrated care models. In China, Li and Wang [43] also highlighted the importance of helping nursing students master cooperation and teamwork skills, and learn how to communicate with both their peers and patients. This dimension’s high average score could be attributed to the widespread adoption of the “problem-oriented” teaching method in China. This approach encourages students to collaborate in groups to analyze and resolve issues, thereby fostering the development of communication, collaboration, and teamwork competencies [44].

The definition of “Patient-centered Care” is referred to as the knowledge, skills, and attitudes of BNSs mastered to provide humanistic care and effective communication with patients, satisfy patient needs, and guide or care patients or their families to involve self-care activities in their future clinical work. Charette, et al. [45] also mentioned this domain in their study. The significance of patient-centered care has grown substantially, as it correlates with enhanced quality of care [46]. Studies have demonstrated that patient satisfaction rises when students exhibit a positive outlook towards patient-centered care [47]. In nursing specialty courses, educators typically underscore the importance of prioritizing patient-centered care. Consequently, this dimension received a relatively high average score. However, the item pertaining to “correctly assessing the physical, spiritual, psychological, cultural, and social needs of patients” scored the lowest. This could be attributed to nursing students’ deficiency in evaluating patients as holistic individuals [48]. Thus, nursing students should be encouraged to carry out holistic and patient-centered nursing care and adapt to the varieties from person to person. Schools or hospital managers should also set up courses or provide an environment to help students develop their comprehensive humanistic and cultural nursing care abilities in their future work.

Informatics competency refers to the knowledge, skills, and attitudes of BNSs in the future clinical work to obtain and utilize information to implement management, provide nursing care, conduct scientific research, and improve the progress of nursing care. The hereby obtained results were in line with those proposed by Nygårdh, et al. [18]. Given the pervasive integration of information technologies into the healthcare system [49], nurses, comprising the majority of the workforce in the health sector, require proficient nursing informatics skills [50]. Chinese nursing educators have developed the information literacy of students by setting up the section of information management in the *Nursing Management* textbook [37]. The item concerning “making correct decisions about relatively complex care issues based on the retrieval of relevant information” received the lowest score. This could be attributed to nursing students’ insufficient mastery of information retrieval skills and their ineffective utilization of health information [51, 52]. Consequently, integrating nursing informatics, particularly for the retrieval of relevant nursing care information, into undergraduate nursing education can enhance nurses’ capability to leverage technology in addressing patients’ complex issues. This, in turn, empowers them to deliver high-quality nursing care.

Evidence-based practice (EBP) competency is defined as the knowledge, skills, and attitudes of BNSs in the future clinical work, which can effectively integrate the clinical opinions of the experts as well as the most effective empirical evidence in clinical nursing care. These findings were consistent with those of the study by Nygårdh, et al. [18], who proposed that it was vital to provide best care on the basis of evidence-based nursing practice. The ability of evidence-based nursing practice can help nursing students solve complicated clinical problems by using evidence instead of subjective judgment in their practice or future work [53, 54]. However, the item regarding “describing the meaning of evidence-based nursing care” obtained the lowest score within this dimension. This could be attributed to EBP being a core course primarily for master’s degree nursing students, with less emphasis on undergraduate education [55]. The findings underscored by Adamakidou et al. [56] highlight the imperative of enhancing nurses’ competence in EBP through education, suggesting that introducing EBP education at the undergraduate level to raise awareness among nursing students is a foundational step. Therefore, nursing educators should impart sufficient knowledge of EBP to bachelor of nursing students and train them to apply EBP principles in resolving patients’ clinical issues.

The definition of continuous quality improvement competency is the knowledge, skills, and attitudes of BNSs in the future clinical work, who are required to be clear about the evaluation criteria and methods of nursing quality, use them to monitor the nursing process, and be able to perform improvement methods to continuously enhance the nursing care quality and safety. Cronenwett, et al. [7] and Nygårdh, et al. [18] also stated the same conclusion. This dimension exhibited the lowest average score among the six dimensions. Notably, the item addressing “describing continuous quality improvement methods such as Deming Circle, Root Cause Analysis, and Quality Control Circle” received the lowest rating within this dimension. This could be attributed to BNSs’ limited clinical experience [57]. Furthermore, the abstract nature of the content related to quality improvement methods might pose challenges for these students in comprehension and application [57]. Additionally, researches have consistently indicated that both students and newly graduated registered nurses tend to score lower on quality improvement compared to other QSEN competencies since the inception of QSEN in 2005 [14, 58]. However, it is crucial to underscore that the advancement of patient safety hinges on the knowledge of quality improvement among clinical frontline staff [59]. Thus, it is necessary for nursing students to master the relevant concepts and measures of quality improvement, such as some nursing quality management methods designed in Chinese nursing management courses, including the plan-do-check-act (PDCA) cycle, root cause analysis (RCA), and clinical pathway (CP) [37]. Besides, cultivating the competency of continuous quality improvement enables nursing students to be aware of the importance of continuous quality improvement as part of their daily work, which may encourage them to get continuously involved in quality improving activities.

## Limitation

However, the present study is still exposed to some limitations. Firstly, this scale was only used to measure the competency of quality and safety regarding greenhand nurses with bachelor degree, and might be limited to assess other levels of greenhand nurses. Therefore, in the subsequent study, greenhand nurses with different educational levels, such as college, can be selected, and the scale can be revised according to their needs. Secondly, given that the participants in this study were not randomly selected, the generalization of this study might be affected.

## Conclusion

CSQS is an effective and valid tool involving 64 items in 6 dimensions to measure the safety and quality competencies of greenhand nurses through CFA testing. Its internal consistency reliability is 0.98 and retest reliability is 0.89, indicating its applicability for the self-evaluation of graduated nursing students or greenhand nurses to find out their deficiencies. In light of the lower-scored items, nursing students can take proactive measures to enhance their safety and quality competencies. Additionally, nursing educators can utilize CSQS to evaluate BNSs, identifying areas of weakness. Subsequently, educators can refine teaching materials, methodologies, or even course curricula to bolster students’ quality and safety competencies. Moreover, hospital nursing managers can employ this tool to thoroughly assess the deficiencies of new nurses and ascertain their precise requirements for safety and quality knowledge. Subsequently, managers can leverage the assessment results as evidence to design targeted clinical safety and quality training programs.

### Electronic supplementary material

Below is the link to the electronic supplementary material.

Supplementary Material 1

## Data Availability

The datasets used and analyzed during the current study are available from the corresponding author upon reasonable request.

## Abbreviations

CFA: Confirmatory Factor Analysis
EFA: Exploratory Factor Analysis
USA: United States of America
CSQS: Competency Scale of Quality and Safety
QSEN: Quality and Safety Education for Nurses
QSEN-SES: Quality and Safety Education for Nurses of student evaluation survey
ICN: International Nursing Council
BNS: Bachelor Nursing Students
QSSI: Quality and Safety Self-Inventory
QSC: Quality and Safety Competencies
CLA: Consensus Level of Agreement
I-CVI: Item Content Validity Index
S-CVI/Ave: Content Validity Index of scale average
ITC: Item-to-Total Correlation
ICR: Internal Consistency Reliability
SD: Standard Deviations
RMSEA: Root Mean Square Error of Approximation
GFI: Goodness of Fit Index
AGFI: Adjusted Goodness of Fit Index
SRMSR: Standardized Root Mean Square Residual
NFI: Normed Fit Index
PCC: Patient-centered care
CAT: Collaboration and Teamwork
EBP: Evidence-based practice
CQI: Continuous quality improvement
SAF: Safety
INF: Informatics
PDCA: Plan-Do-Check-Act
RCA: Root Cause Analysis
CP: Clinical Pathway
WHO: World Health Organization
