# Supplementary material for: Competency scale of quality and safety for greenhand nurses: instrument development and psychometric test
Source: BMC Nurs. 2024 Mar 29;23:219. doi: 10.1186/s12912-024-01873-5 (PMC10979626; doi:10.1186/s12912-024-01873-5)
Supplement: Supplementary file 1 — Supplementary Material 1 [file 12912_2024_1873_MOESM1_ESM.pdf]

Additional Table 1 Item's description and factor loading regarding the Competency Scale of Quality and Safety (N = 1221)

Domains	Latent constructs	b	B	SE	t-value	R^2	Error	Mean	SD	Min	Max	Median
1. Patient-centered care	1) Identify the benefits of patients' involvement in health care for their own health education decisions.(Knowledge)	1.00	0.59			0.35	0.65	3.84	0.797	1	5	4.00
	2) Understand humanistic care in nursing. (Knowledge)	0.94	0.59	0.05	20.36*	0.35	0.65	3.94	0.742	2	5	4.00
	3) Understand the way to build a nurse-patient relationship with patients. (Knowledge)	1.05	0.67	0.05	20.00*	0.45	0.55	3.95	0.736	1	5	4.00
	4) Understand the skills of and obstacles to effectively communicating with patients. (Knowledge)	1.05	0.66	0.05	20.35*	0.44	0.56	3.78	0.749	1	5	4.00
	5) Guide patients in expressing their wishes, requests or preferences during conversations with them. (Skill)	1.14	0.71	0.06	20.31*	0.50	0.50	3.82	0.760	2	5	4.00
	6) Correctly assess the physical, spiritual, psychological, cultural and social needs of patients. (Skill)	1.19	0.70	0.06	19.51*	0.49	0.51	3.65	0.800	1	5	4.00
	7) Persuade patients to change those lifestyle habits detrimental to their health from the health perspective. (Skill)	1.17	0.69	0.06	19.24*	0.48	0.52	3.69	0.799	1	5	4.00
	8) Encourage patients or their families to actively participate in self-management	1.18	0.72	0.06	19.91*	0.52	0.48	3.83	0.764	1	5	4.00

	of health promotion. (Skill)											
	9) Use humanistic skills in the provision of care to patients. (Skill)	1.21	0.75	0.06	20.53*	0.57	0.43	3.85	0.751	1	5	4.00
	10) Communicate effectively with patients and their families. (Skill)	1.09	0.72	0.06	19.47*	0.52	0.48	3.89	0.709	1	5	4.00
	11) Value the needs or views of patients in their personal health and illness.(Attitude)	1.11	0.73	0.06	20.01*	0.53	0.47	3.94	0.718	2	5	4.00
	12) Recognize the importance of active involvement of patients and their families in the development and implementation of care plans as well as the evaluation of outcomes. (Attitude)	1.10	0.70	0.06	19.35*	0.49	0.51	3.81	0.738	1	5	4.00
	13) Focus on continually improving communicating and conflict resolving skills. (Attitude)	1.17	0.74	0.06	20.34*	0.55	0.45	3.80	0.737	1	5	4.00
2. Collaboration and Teamwork	14) Understand the important role of team members, including doctors, head nurses, ward nurses, caregivers, physical therapists, family members, housekeepers, etc., in providing continuous care to main the sense of safety of patients. (Knowledge)	1.00	0.65			0.43	0.57	3.97	0.753	1	5	4.00
	15) Identify the weaknesses and strengths in daily work to achieve team goals. (Knowledge)	1.00	0.71	0.04	23.89*	0.50	0.50	3.91	0.693	2	5	4.00

16) Present examples of the impact of teamwork on the quality and safety care of patients (e.g., collaboration between doctors, carers, ward nurses, family members, housekeepers, physical therapists, etc.). (Knowledge)	1.08	0.72	0.04	23.96*	0.52	0.48	3.80	0.733	1	5	4.00
17) Describe the impact of one's personal communicating style on others. (Knowledge)	1.06	0.71	0.05	22.12*	0.51	0.49	3.74	0.722	1	5	4.00
18) Discuss effective strategies for conflict resolution and effective communication. (Knowledge)	1.08	0.69	0.05	21.39*	0.47	0.53	3.65	0.775	1	5	4.00
19) Integrate reasonable advice or suggestions from family members when helping patients and encourage active participation of team members. (Skill)	1.10	0.73	0.05	21.84*	0.53	0.47	3.76	0.734	1	5	4.00
20) Communicate effectively with different team members using appropriate methods.(Skill)	1.07	0.72	0.05	22.22*	0.52	0.48	3.75	0.729	1	5	4.00
21) Meet commitments of team goals. (Skill)	1.06	0.72	0.05	22.17*	0.51	0.49	3.84	0.720	2	5	4.00
22) Enlist the input of other team members to improve the overall team performance. (Skill)	1.11	0.70	0.05	20.84*	0.49	0.51	3.70	0.775	1	5	4.00
23) Minimize risks between caregivers and in patient transfers (e.g., patient	1.12	0.74	0.05	23.01*	0.55	0.45	3.76	0.730	1	5	4.00

	communication in transfers) following principles of communication practice. (Skill)											
	24) Value the views, expertise or opinions of all team members. (Attitude)	1.00	0.65	0.05	20.36*	0.42	0.58	3.87	0.751	1	5	4.00
	25) Respect the different communication styles of team members. (Attitude)	0.96	0.64	0.05	20.20*	0.41	0.59	3.97	0.734	1	5	4.00
	26) Recognize the importance of collaboration within the nursing profession or among interdisciplinary professions. (Attitude)	0.94	0.62	0.05	20.30*	0.38	0.62	3.96	0.747	1	5	4.00
	27) Focus on completing individual responsibilities in a collaborative team. (Attitude)	1.06	0.72	0.05	22.00*	0.51	0.49	3.97	0.726	1	5	4.00
3. Evidence-based practice	28) Master basic scientific research methods in their undergraduate studies. (Knowledge)	1.00	0.67			0.45	0.55	3.67	0.809	1	5	4.00
	29) Distinguish correctly between clinical nursing experience and the results of scientific research. (Knowledge)	1.06	0.71	0.04	26.57*	0.51	0.49	3.69	0.800	1	5	4.00
	30) Describe the meaning of evidence-based nursing care. (Knowledge)	1.08	0.68	0.04	24.98*	0.46	0.54	3.55	0.862	1	5	4.00
	31) Explain the positive role of evidence-based nursing practice in	1.06	0.70	0.04	24.57*	0.49	0.51	3.76	0.812	1	5	4.00

	"patient-centered" care. (Knowledge)											
	32) Consult clinical experts in an appropriate and timely manner in the case of problems in clinical practice. (Skill)	1.06	0.71	0.05	22.23*	0.50	0.50	3.71	0.803	1	5	4.00
	33) Attach importance to the required moral and ethical principles in the process of scientific research. (Attitude)	0.99	0.70	0.05	20.75*	0.49	0.51	3.81	0.758	2	5	4.00
	34) Value the importance of regular reading of relevant professional journals. (Attitude)	1.06	0.71	0.55	22.35*	0.50	0.50	3.69	0.805	1	5	4.00
4. Continuous quality improvement	35) Recognize the essential role of nursing students in the healthcare system and the nursing process as well as the impact of their participation on the care outcomes of patients. (Knowledge)	1.00	0.68			0.46	0.54	3.78	0.780	1	5	4.00
	36) Describe continuous quality improvement methods such as Deming Circle, Root Cause Analysis and Quality Control Circle. (Knowledge)	1.14	0.60	0.06	19.49*	0.36	0.64	3.23	1.016	1	5	3.00
	37) Clarify clinical criteria for evaluating the care quality (e.g., criteria for evaluating the quality of writing nursing documents). (Knowledge)	1.12	0.72	0.05	23.05*	0.52	0.48	3.53	0.821	1	5	4.00
	38) Apply at least one quality evaluation method to the assessment of the	1.10	0.72	0.05	22.87*	0.51	0.49	3.54	0.819	1	5	4.00

	effectiveness of holistic care. (Skill)											
	39) Accept continuous quality improvement as an important part of the daily work of all health workers. (Attitude)	1.03	0.70	0.05	22.15*	0.49	0.51	3.75	0.779	1	5	4.00
	40) Value the use of quality evaluation tools in quality care services.(Attitude)	1.00	0.70	0.04	22.32*	0.49	0.51	3.70	0.756	1	5	4.00
	41) Enjoy continuous improvement in the quality of patient care in daily nursing practice. (Attitude)	0.96	0.68	0.04	21.73*	0.47	0.53	3.82	0.751	1	5	4.00
5. Safety	42) Master the technical specifications of nursing operations. (Knowledge)	1.00	0.71			0.51	0.49	3.88	0.755	1	5	4.00
	43） Master all hospital nursing regulations and rules. (Knowledge)	0.99	0.70	0.03	28.63*	0.49	0.51	3.95	0.763	1	5	4.00
	44) Consciously comply with hospital regulations and rules in their work and regulate their personal nursing behaviors. (Skill)	0.89	0.63	0.04	25.24*	0.40	0.60	4.02	0.772	1	5	4.00
	45) Communicate with other team members about potential events they are aware of or observe to cause harm to patients. (Skill)	0.98	0.71	0.04	24.82*	0.51	0.49	3.93	0.735	1	5	4.00
	46) Handle specialist resuscitation procedures and be capable of performing resuscitation work. (Skill)	1.12	0.70	0.05	23.13*	0.49	0.51	3.59	0.858	1	5	4.00
	47) Proactively report and actively	1.04	0.74	0.04	25.04*	0.54	0.46	3.85	0.754	1	5	4.00

	participate in the analysis of reasons instead of complaining about nursing deficiencies. (Skill)											
	48) Pay attention to strengthening the standardization of their own technical practices to ensure safe patient care. (Attitude)	1.02	0.73	0.04	24.51*	0.53	0.47	3.88	0.754	1	5	4.00
	49) Focus on proactive reporting of safety accidents, hazards, potential failures and adverse events. (Attitude)	1.10	0.77	0.04	24.96*	0.59	0.41	3.87	0.770	1	5	4.00
	50) Identify the sources of occupational hazards for nurses. (Knowledge)	0.94	0.68	0.04	22.81*	0.46	0.54	3.95	0.741	1	5	4.00
	51) Focus on the prevention of occupational risk factors in the workplace. (Attitude)	0.97	0.71	0.04	24.26*	0.50	0.50	3.94	0.738	1	5	4.00
	52) Take effective occupational protection measures to minimize occupational hazards in clinical practice. (Skill)	0.96	0.72	0.04	24.02*	0.51	0.49	3.92	0.723	1	5	4.00
6. Informatics	53) Handle reliable medical information resources. (Knowledge)	1.00	0.70			0.49	0.51	3.73	0.766	1	5	4.00
	54) Focus on information management strategies to ensure patient safety. (Knowledge)	0.99	0.70	0.04	26.74*	0.49	0.51	3.74	0.750	1	5	4.00
	55) Introduce the basis of retrieving nursing information. (Knowledge)	1.05	0.70	0.04	25.64*	0.49	0.51	3.64	0.802	1	5	4.00

56) Understand how information technology (IT) tools can support patient safety management (e.g., use of electronic information systems for medical orders). (Skill)	1.10	0.73	0.05	23.28*	0.54	0.46	3.71	0.795	1	5	4.00
57) Understand the role of information management tools efficiency in effectively monitoring care delivery (e.g., reporting of nursing deficiencies such as pressure sores, falls, etc.). (Skill)	1.09	0.71	0.05	23.30*	0.51	0.49	3.72	0.809	1	5	4.00
58) Handle the skills to meet the nursing needs via IT approaches (e.g., entering nursing records using the hospital electronic information system). (Skill)	1.07	0.71	0.05	23.42*	0.51	0.49	3.77	0.794	1	5	4.00
59) Document the patient care process using an electronic information collection system.(Skill)	1.07	0.72	0.05	23.72*	0.52	0.48	3.77	0.787	1	5	4.00
60) Make correct decisions about relatively complex care issues based on the retrieval of relevant information. (Skill)	1.05	0.69	0.05	23.26*	0.48	0.52	3.61	0.810	1	5	4.00
61) Identify the need for continuous lifelong IT learning of all nursing staff. (Attitude)	1.00	0.67	0.05	21.29*	0.45	0.55	3.94	0.796	1	5	4.00
62) Understand the role of IT in the clinical decision-making, coordination	1.00	0.70	0.04	22.99*	0.49	0.51	3.86	0.765	1	5	4.00

and prevention of patient errors.(Attitude)											
63) Protect the confidentiality of electronic information records. (Attitude)	0.95	0.65	0.05	20.96*	0.43	0.57	4.00	0.779	1	5	4.00
64) Gather as much patient information as possible to assist doctors in their diagnosis and treatment. (Attitude)	0.93	0.67	0.04	21.90*	0.45	0.55	3.93	0.753	1	5	4.00

Note: SE= Standard Error, SD= standard deviation, Min=Minimum, Max=Maximum
